# Operational definition of precipitated opioid withdrawal

**DOI:** 10.3389/fpsyt.2023.1141980

**Published:** 2023-04-20

**Authors:** Kelly E. Dunn, H. Elizabeth Bird, Cecilia L. Bergeria, Orrin D. Ware, Eric C. Strain, Andrew S. Huhn

**Affiliations:** ^1^Johns Hopkins University School of Medicine, Baltimore, MD, United States; ^2^University of North Carolina at Chapel Hill School of Social Work, Chapel Hill, NC, United States

**Keywords:** opioids, opioid use disorder, fentanyl, buprenorphine, precipitated, withdrawal, heroin, naloxone

## Abstract

**Background:**

Opioid withdrawal can be expressed as both a spontaneous and precipitated syndrome. Although spontaneous withdrawal is well-characterized, there is no operational definition of precipitated opioid withdrawal.

**Methods:**

People (*N* = 106) with opioid use disorder maintained on morphine received 0.4 mg intramuscular naloxone and completed self-report (Subjective Opiate Withdrawal Scale, SOWS), visual analog scale (VAS), Bad Effects and Sick, and observer ratings (Clinical Opiate Withdrawal Scale, COWS). Time to peak severity and minimal clinically important difference (MCID) in withdrawal severity were calculated. Principal component analysis (PCA) during peak severity were conducted and analyzed with repeated measures analyses of variance (ANOVA).

**Results:**

Within 60 min, 89% of people reported peak SOWS ratings and 90% of people had peak COWS scores as made by raters. Self-reported signs of eyes tearing, yawning, nose running, perspiring, hot flashes, and observed changes in pupil diameter and rhinorrhea/lacrimation were uniquely associated with precipitated withdrawal. VAS ratings of Bad Effect and Sick served as statistically significant severity categories (0, 1–40, 41–80, and 81–100) for MCID evaluations and revealed participants' identification with an increase of 10 [SOWS; 15% maximum percent effect (MPE)] and 6 (COWS; 12% MPE) points as meaningful shifts in withdrawal severity indicative of precipitated withdrawal.

**Conclusion:**

Data suggested that a change of 10 (15% MPE) and 6 (12% MPE) points on the SOWS and COWS, respectively, that occurred within 60 min of antagonist administration was identified by participants as a clinically meaningful increase in symptom severity. These data provide a method to begin examining precipitated opioid withdrawal.

## 1. Introduction

People who consume opioids chronically often develop opioid physical dependence and a corresponding opioid withdrawal syndrome (1, 2). The manifestation of opioid withdrawal is hypothesized to be driven, in part, by a deficit in mu-opioid receptor occupancy resulting from a reduction in exogenously administered opioid agonists (e.g., heroin and fentanyl) or endogenous opioid signaling as a function of receptor downregulation (2). The opioid withdrawal syndrome etiology and severity can be easily evaluated via self-reported and/or observer rating scales (3), and two forms of the opioid withdrawal syndrome are currently recognized. The first is spontaneous opioid withdrawal, which is the more common clinical condition that has been well-characterized and is often a major focus for medications for opioid use disorder (MOUDs). Spontaneous opioid withdrawal symptoms begin to emerge as soon as 4–6 h after the last opioid dose was consumed and increase in severity in a linear fashion over a several-day period before peaking and remitting (3, 4).

The second opioid withdrawal syndrome, precipitated opioid withdrawal, has not been thoroughly characterized. In contrast to spontaneous withdrawal, which emerges naturally following a period of opioid abstinence, precipitated withdrawal is elicited through the provision of an opioid antagonist (naloxone and naltrexone) or buprenorphine (a mu-opioid partial receptor agonist with a low ceiling on its effects) that is administered to an individual who is physically dependent on opioids and/or in close proximity to an opioid agonist (5). Precipitated opioid withdrawal manifests in a very different time course relative to spontaneous withdrawal, often emerging, peaking, and fully remitting within a short-circumscribed period (the length of which depends upon the half-life of the antagonist administered). For instance, naloxone-precipitated withdrawal largely resolves within 120 min following naloxone administration (6).

There currently exists no operational definition of precipitated opioid withdrawal. This is problematic as there are several clinical settings in which precipitated opioid withdrawal may occur, most notably the provision of naloxone for reversal of agonist effects (e.g., overdose reversal), but also the transition of a patient from opioid agonists onto the MOUDs naltrexone or buprenorphine. A lack of operational definition challenges clinical care for people with OUD because people seeking treatment with buprenorphine or naltrexone are instructed to abstain from opioid agonists for hours (buprenorphine) or days (naltrexone) before induction (5). Thus, symptoms of withdrawal in these patients could represent either emergent spontaneous opioid withdrawal as a result of recent abstinence or precipitated opioid withdrawal symptoms as a result of the MOUD administration. The lack of an operational definition precludes the determination of the origin of the syndrome and obscures potential remediation strategies.

The goal of these analyses was to develop a working operational definition of precipitated opioid withdrawal. Analyses are derived from a secondary analysis of known naloxone-precipitated withdrawal in people maintained on the short-acting opioid morphine. Outcomes focused on the identification of the threshold of change in withdrawal severity that best indicated the precipitated opioid withdrawal, as reflected by a change in syndrome expression and subjective determination of change in withdrawal status by the patients.

## 2. Methods

### 2.1. Participants

Participants (*N* = 106) were people with OUD who were enrolled in a residential randomized clinical trial comparison of clonidine, tramadol-extended release, and buprenorphine for supervised opioid withdrawal between October 2010 and June 2015 [see Dunn et al. (7)]. Eligible participants were aged 18–60, met DSM-IV criteria for opioid dependence, provided a urine sample that tested positive for opioids or showed evidence of opioid withdrawal, and had no significant medical or psychiatric illness. Participants were excluded if pregnant, had benzodiazepine or alcohol physical dependence, hypotension, a history of seizures, known allergy to study medications, or were currently enrolled in opioid agonist treatment. All participants provided voluntary informed consent to participate in the trial. Only methods relevant to the following analyses are presented here.

### 2.2. Study design

Before randomization, all participants were maintained on morphine (30 mg, subcutaneous, QID) for up to 10 days and completed one naloxone challenge test after 4 days of morphine stabilization. On the challenge day, participants received morphine at 07:00, completed a series of baseline withdrawal ratings (described below) at 10:45, and received a 0.4 mg intramuscular naloxone injection at 11:00. Withdrawal ratings were repeated at 15-min intervals for 120 min following naloxone administration, at which time participants received morphine to remediate any residual withdrawal.

### 2.3. Withdrawal ratings

Self-reported withdrawal was collected using the Subjective Opiate Withdrawal Scale [SOWS (8)], a 16-item measure on which participants rate the severity of their opioid withdrawal symptoms on a 0–4 Likert scale (range 0–64) and Visual Analog Scale (VAS) ratings of “Bad Effects” and “Sick” rated on scales of 0 (not at all) to 100 (extremely). Observer ratings were collected using the Clinical Opioid Withdrawal Scale [COWS (9)], an 11-item clinician-administered measure that uses different ordinal scales for each symptom (range 0–48).

### 2.4. Data strategy

The analyses examined an (1) optimal time period in which to observe precipitated withdrawal and (2) the threshold of change in withdrawal score required before participants identified it as representing a change in withdrawal severity. SOWS and COWS total scores were converted to change from baseline by subtracting out the pre-naloxone score for use as primary outcome variables. The time period in which withdrawal was observed was described and evaluated by calculating the time that peak SOWS and COWS change scores were detected for each participant, which was examined using one-way analyses of variance (ANOVA).

Next, withdrawal severity was identified by calculating the minimal clinically important difference (MCID) utilizing the anchor method, which requires a concurrent rating of general patient status against which the withdrawal ratings of interest can be compared (10). The MCID approach was preferable for these analyses because it utilizes continuous outcomes (e.g., VAS) to identify the smallest difference in a score that participants can perceive as representing a clinically important change in their status. The VAS ratings of Bad Effects and Sick were examined as anchor variables (i.e., external standards against which the variables of interest can be compared) because they were collected concurrent with the SOWS and COWS and represented global measures of patient status (vs. symptom-driven measures which may not capture the global experience). The internal validity of the Bad Effects and Sick ratings was examined using area under the curve via receiver operating characteristic (ROC) curves and one-way ANOVAs, as outlined below. Based on these analyses, Bad Effect and Sick ratings were then categorized into ordinal scales that represented increasing severity levels and used as anchors against which the SOWS and COWS MCID thresholds could be derived. MCIDs were calculated as outlined below and examined for internal consistency by calculating their positive predictive values (PPV) to assess their precision for identifying changes in withdrawal severity as defined by the VAS Bad Effect and Sick scales. Finally, principal component analyses (PCA) were conducted on the SOWS and COWS collected at the time of individual participant peak severity to inform whether the precipitated withdrawal was characterized by a unique symptom profile at the time of peak expression. All analyses were conducted using SPSS v. 28, and the alpha was set at 0.05.

## 3. Results

### 3.1. Participant sample

Participants (*N* = 106) were 58.5% Black, 15.1% female, and a mean of 40.6 (SD = 10.3) years of age. They reported using heroin and commercially produced opioids a mean (SD) of 24.8 (8.6) and 2.7 (7.4) days in the past 30 days, respectively, and had been using these drugs regularly for 11.6 (9.7) and 1.5 (3.7) years, respectively. Additional demographics are described elsewhere (7). Withdrawal ratings taken 15 min before the administration of naloxone revealed that participants were in only mild spontaneous withdrawal, as rated by the mean (SD) of the SOWS (2.4 + 3.0, range 0–16) and COWS (1.5 + 1.7, range 0–9).

### 3.2. Time course

**Figure 1:**
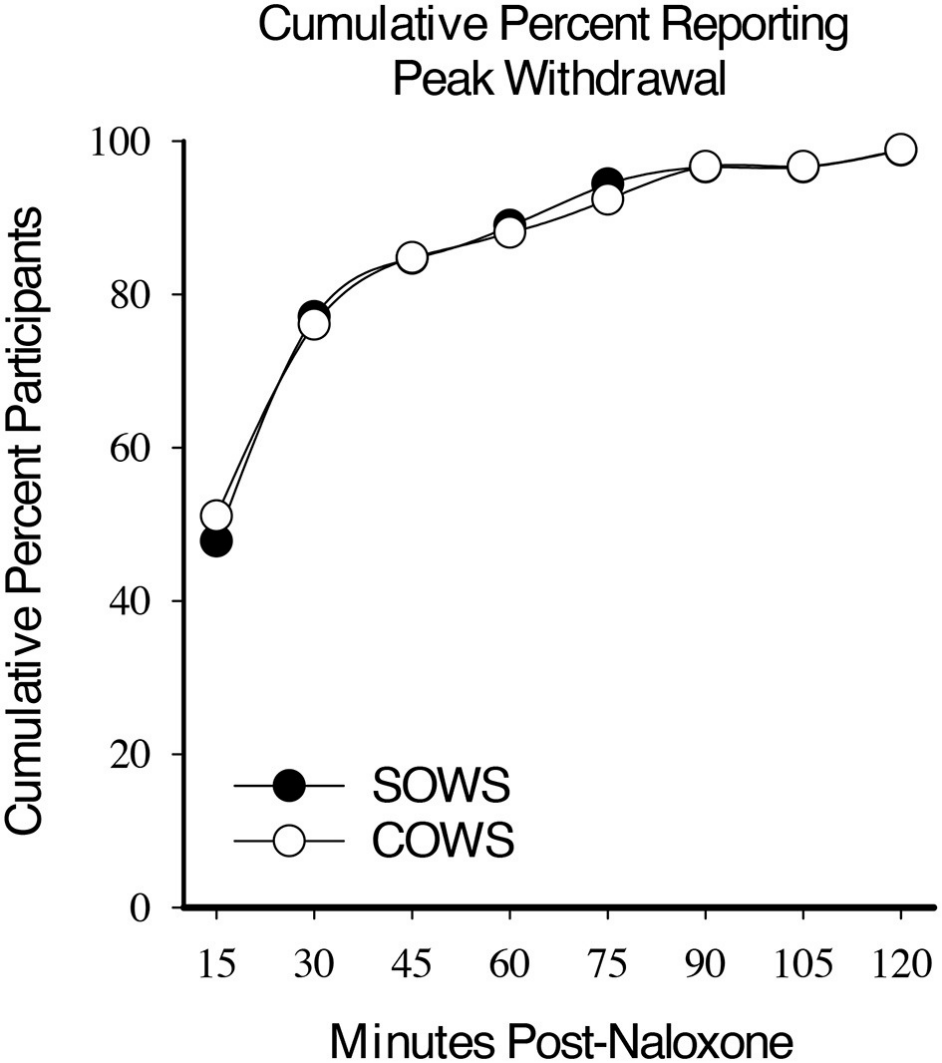
Cumulative peak experience. Cumulative percentage of participants who reported peak self-reported (Subjective Opiate Withdrawal Scale; SOWS, filled circle) and observer-rated (Clinical Opiate Withdrawal Scale; COWS, open circles) scores at each 15-min timepoint post-naloxone (0.4 mg intramuscular dose).

Within-subject analyses revealed a significant main effect for time on the SOWS [*F*_(8, 632)_ = 57.8, *p* < 0.001] and COWS [*F*_(8, 632)_ = 68.3, *p* < 0.001] total scores for all post-administration timepoints. Approximately half the participants demonstrated peak ratings for the SOWS (51%) and COWS (48%) within 15 min. By 30 min, a cumulative 76% (SOWS) and 78% (COWS) of participants had reached peak ratings. Within 60 min, 89 and 90% of all participants had reported peak ratings on the SOWS and COWS, respectively (Figure 1).

### 3.3. VAS scales as anchor variables

VAS ratings of Bad Effects and Sick were dichotomized as representing no effects (score 0–19) or any effect (20–100) at each post-naloxone timepoint and examined using ROC curve analyses, with an area under the curve (AUC) of >0.70 set as an *a priori* definition of good fit. Using this approach, VAS Bad Effect ratings yielded AUC values of 0.775 (*p* < 0.01) and 0.758 (*p* < 0.01) for the SOWS and COWS, respectively. VAS Sick ratings yielded similar AUC values of 0.764 (*p* < 0.01) and 0.746 (*p* < 0.01) for the SOWS and COWS, respectively.

**Figure 2:**
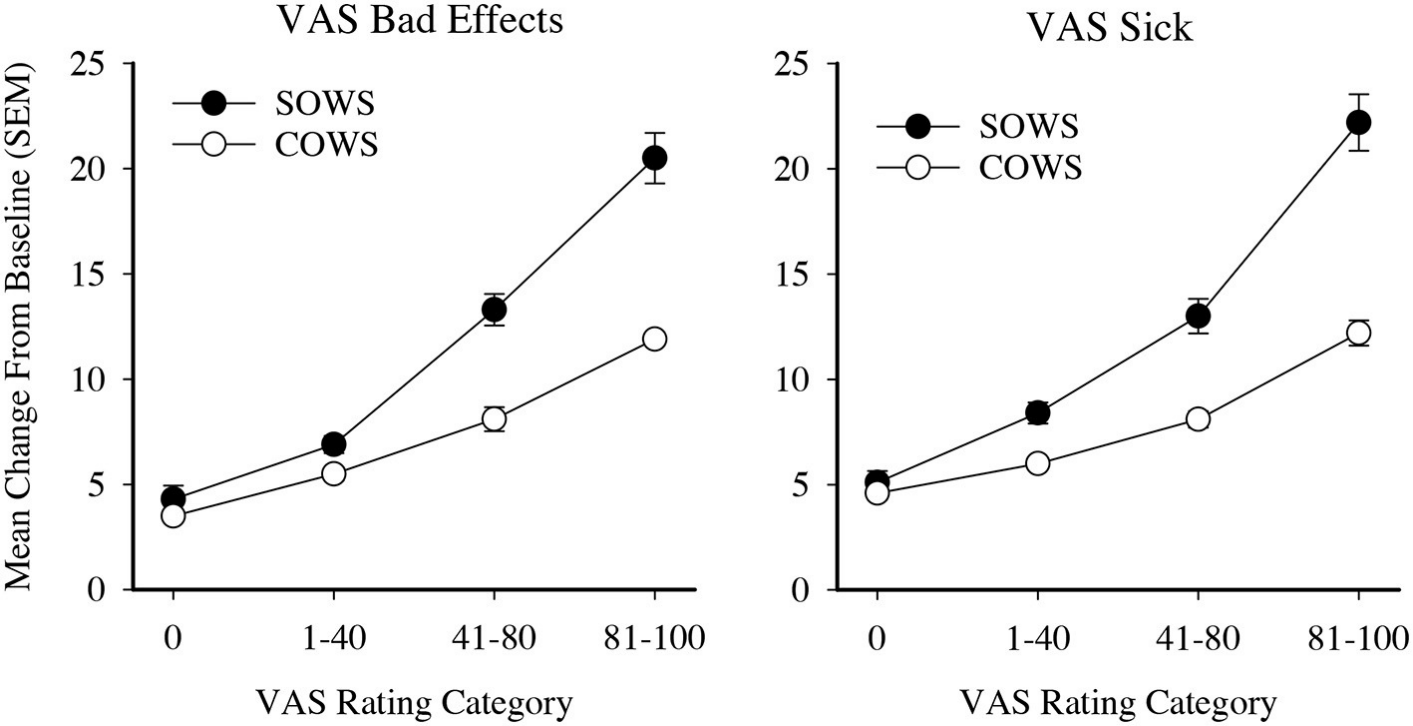
Visual analog ratings (VAS) as severity scores. Data present severity categories for the self-reported Subjective Opiate Withdrawal Scale (SOWS, filled circles) and observer-rated Clinical Opiate Withdrawal Scale (COWS, open circles). All data points are significantly different from each other within each scale and outcome examined.

VAS ratings were then examined for clinical relevance. Ratings were initially categorized into five severity thresholds (score ranges 0–20, 21–40, 41–60, 61–80, and 81–100) and used as an independent variable to examine SOWS and COWS change from baseline ratings in one-way ANOVAs with Tukey *post hoc* tests. Analyses for the SOWS and COWS were significant for both the Bad Effects [*F*_(4, 691)_ = 66.1, *p* < 0.001, eta^2^ = 0.28 and *F*_(4, 689)_ = 65.4, *p* < 0.001, eta^2^ = 0.28, respectively] and Sick [*F*_(4, 691)_ = 62.2, *p* < 0.001, eta^2^ = 0.27 and *F*_(4, 689)_ = 52.0, *p* < 0.001, eta^2^ = 0.23, respectively] scales; however, *post hoc* analyses revealed substantial statistical overlap for ratings within the 0–40 and 41–80 ranges. VAS ratings were then reclassified into four categories (0, 1–40, 41–80, and 81–100) and reanalyzed using one-way ANOVAs. Results remained significant for the SOWS and COWS on VAS Bad Effects [*F*_(3, 691)_ = 90.2, *p* < 0.001, eta^2^ = 0.28 and *F*_(3, 689)_ = 92.2, *p* < 0.001, eta^2^ = 0.29, respectively] and Sick [*F*_(3, 691)_ = 85.0, *p* < 0.001, eta^2^ = 0.27 and *F*_(3, 689)_ = 65.6, *p* < 0.001, eta^2^ = 0.22, respectively] scales, and *post hoc* testing revealed that all severity levels were significantly different from one another within each scale (Figure 2). Based on these data, both the 4-level Bad Effect and Sick scales were retained for MCID examinations.

### 3.4. MCID

The MCID or the mean increase in withdrawal ratings that prompted participants to increase their VAS ratings by ≥1 severity threshold (e.g., the aforementioned MCID ratings of 0, 1–40, 41–80, and 81–100) was calculated as a measure of onset of clinically significant precipitated withdrawal. The MCIDs for the Bad Effects severity rating were 10.5 (SOWS) and 6.5 (COWS) and for the Sick severity rating were 10.3 (SOWS) and 6.7 (COWS). The SOWS and COWS change from baseline scores were then dichotomized at 10 (SOWS) and 6 (COWS). To support generalizability, scores were also converted into maximum percent effect, representing a change of 15% (SOWS) and 12% (COWS).

The SOWS and COWS thresholds were then examined for their positive predictive value (PPV), which is defined here as the percentage of participants who had a change in SOWS or COWS at or above the stated thresholds who did not rate their Bad Effects and/or Sick scales as 0. As evident in Table 1, the SOWS and COWS precipitated withdrawal thresholds of 10 and 6 yielded a low rate of false positives (5.3% and 4.2% of ratings, respectively) when the standard was VAS Bad Effects. The false positive rate was higher (11.1% and 13.0%, respectively) when the standard was VAS Sick. Additional cut-points (outlined in Table 1) that were assessed for both the SOWS and COWS suggested sensitivity increased (and the rate of false positives decreased) as the change score increased.

**Table 1:** Minimal clinically important different (MCID) to predict change in VAS severity categories.

**Scale**	**MCID: increase from baseline (# points)**	**Positive predictive value**							
		**VAS bad effects: 0**	***N***	**VAS bad effects: 1–40**	***N***	**VAS bad effects: 41–80**	***N***	**VAS bad effects: 81–100**	***N***
		**% (95% CI)**		**% (95% CI)**		**% (95% CI)**		**% (95% CI)**	
SOWS	8	5.1 (0.0–16.2)	14	20.7 (10.2–31.3)	57	33.7 (24.1–43.3)	93	40.6 (31.5–49.7)	112
	9	4.8 (0.0–16.9)	12	18.7 (10.2–31.2)	47	33.7 (23.7–43.7)	85	42.9 (33.6–52.2)	108
	10	5.3 (0.0–18.0)	12	15.5 (3.5–27.5)	35	33.6 (23.0–44.2)	76	45.6 (36.0–55.2)	103
	11	4.7 (0.0–17.8)	10	15.2 (2.7–27.6)	32	33.2 (22.2–44.2)	70	46.9 (36.0–55.2)	99
	12	4.1 (0.0–17.8)	8	13.2 (0.0–26.2)	26	34.0 (22.7–45.3)	67	48.7 (38.7–56.7)	96
COWS	4	7.5 (0.0–17.1)	29	27.8 (19.4–36.2)	108	31.2 (22.9–39.4)	121	33.5 (25.4–41.2)	130
	5	6.1 (0.0–16.5)	20	24.8 (15.4–34.2)	81	31.9 (22.9–40.1)	104	37.1 (28.5–45.7)	121
	6	4.2 (0.0–15.5)	12	22.5 (12.3–32.7)	64	33.7 (24.2–43.1)	96	39.6 (30.1–48.6)	113
	7	3.3 (0.0–15.7)	8	19.6 (9.3–30.1)	48	34.7 (24.5–44.8)	85	42.4 (32.9–51.9)	104
	8	2.4 (0.0–15.8)	5	18.4 (6.1–30.1)	38	32.0 (20.7–43.3)	66	47.1 (37.2–57.0)	97
		**VAS sick: 0**	***N***	**VAS sick: 1–40**	***N***	**VAS sick: 41–80**	***N***	**VAS sick: 81–100**	***N***
		**% (95% CI)**		**% (95% CI)**		**% (95% CI)**		**% (95% CI)**	
SOWS	8	12.3 (1.0–23.3)	34	29.0 (19.1–40.0)	80	25.7 (15.6–35.8)	71	33.0 (23.3–42.6)	91
	9	11.9 (0.0–23.4)	30	26.2 (15.6–36.8)	66	26.2 (15.6–36.8)	66	35.7 (25.8–45.6)	90
	10	11.1 (0.0–23.4)	25	24.3 (12.9–35.6)	66	26.5 (15.3–37.7)	60	38.1 (27.8–48.3)	86
	11	11.4 (0.0–24.1)	24	23.2 (11.4–35.0)	49	26.5 (14.9–38.1)	56	38.9 (28.3–49.5)	82
	12	10.7 (2.0–23.9)	21	21.3 (8.9–33.6)	42	27.4 (15.5–39.2)	54	40.6 (29.8–51.3)	80
COWS	4	14.9 (5.7–24.1)	58	32.5 (24.3–40.1)	126	25.3 (16.7–33.9)	98	27.3 (18.8–35.8)	106
	5	14.1 (4.0–24.1)	46	28.5 (19.3–37.7)	93	26.7 (17.4–36.0)	87	30.7 (21.7–39.7)	100
	6	13.0 (2.2–23.8)	37	24.9 (14.8–35.0)	71	28.4 (18.6–38.2)	81	33.7 (24.2–43.2)	96
	7	11.0 (0.0–22.8)	27	24.9 (14.0–35.7)	61	27.8 (17.2–38.4)	68	36.3 (26.3–46.2)	89
	8	11.0 (0.0–24.1)	23	22.3 (10.2–34.3)	46	25.7 (13.9–37.5)	53	40.8 (30.2–51.3)	83

CI, confidence interval; COWS, Clinical Opiate Withdrawal Scale; SOWS, Subjective Opiate Withdrawal Scale; VAS, Visual Analog Scale.

### 3.5. Principal components analysis of symptoms

Symptom ratings at the time of peak withdrawal for each participant were evaluated for the SOWS and COWS separately using PCA with a Varimax rotation. SOWS symptoms are rated on the same 0–4 Likert scale and were analyzed as a function of the raw value. COWS symptoms are rated on different ordinal scales, and were converted to *Z* scores for analyses. Factors were defined by an eigenvalue of >1.0 and individual symptoms with a rotated factor loading of >0.50 were retained. When PCAs were conducted without constraining the number of factors, the SOWS yielded a three-factor structure and the COWS yielded a four-factor structure. However, not all factors were meaningful (e.g., had <2 items loaded). The PCAs were then re-run until ≥2 items were satisfactorily loaded onto a single factor, resulting in a two-factor structure for each scale.

**Figure 3:**
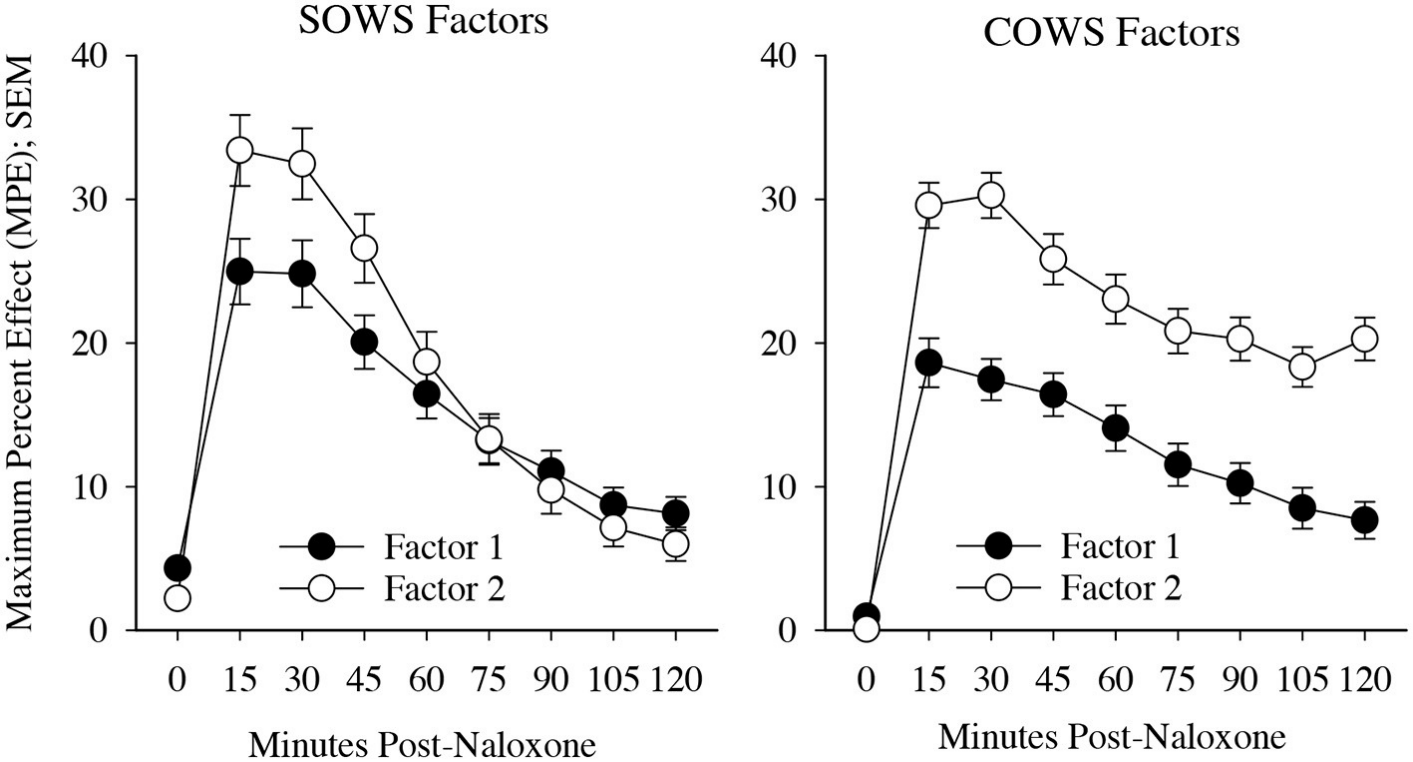
Precipitated withdrawal symptoms. Change in factors for the Subjective Opiate Withdrawal Scale (SOWS, left) and Clinical Opiate Withdrawal Scale (COWS, right) over time (*X*-axis; standard error of the mean [SEM]). Factors were derived using principal component analyses based on symptom severity ratings collected at the time of peak withdrawal following 0.4 mg (intramuscular) naloxone dosing. Scales are converted to maximum percent effect (MPE) along *Y*-axis for comparison. SOWS Factor 1 included 11 symptoms (nausea, vomiting, restlessness, anxiety, shaking, muscle twitching, stomach cramps, feel like using, cold flashes, gooseflesh, and bone and muscle aches) and SOWS Factor 2 included five symptoms (eyes tearing, yawning, nose running, perspiring, and hot flashes). COWS Factor 1 included seven symptoms (gastrointestinal upset, tremor, restlessness, anxiety or irritability, bone or joint aches, gooseflesh, and yawning), and COWS Factor 2 included two symptoms (pupil size and rhinorrhea/lacrimation). Two COWS symptoms (resting pulse rate and sweating) did not load on any factor.

The SOWS PCA (Table 2) conducted using symptoms at the time of peak report for each individual was significant [*X*^2^(120) = 1,006.62, *p* < 0.001] and demonstrated excellent sampling adequacy (0.878), with two factors identified and all symptoms loading onto a factor. Factor 1 explained 33.1% of the variance and included the following 11 symptoms: nauseous, vomiting, stomach cramps, bone and muscle aches, gooseflesh, shaking, cold flashes, restlessness, anxiety, feel like using, and muscle twitching. Factor 2 explained 26.9% of the variance and included the following five symptoms: yawning, lacrimation, rhinorrhea, perspiring, and hot flashes. A repeated measure ANOVA revealed significant main effects of factor [*F*_(1, 100)_ = 14.92, *p* < 0.001, eta^2^ = 0.13], time [*F*_(3, 300)_ = 25.61, *p* < 0.001, eta^2^ = 0.20], and a significant factor x time interaction [*F*_(3, 300)_ = 7.86, *p* < 0.001, eta^2^ = 0.07], with the five-item factor 2 demonstrating more severe symptomatology than the 11-item factor 1 at 15, 30, and 45-min post-dosing (see Figure 3).

**Table 2:** Peak precipitated withdrawal symptom factors.

**Scale**	**Factor 1 symptoms**	**Loading value**	**Factor 2 symptoms**	**Loading value**	**Did not load**
Subjective Opiate Withdrawal Scale (SOWS)	Nausea	0.846	Yawning	0.847	–
	Vomiting	0.807	Eyes tearing (lacrimation)	0.838	–
	Stomach cramps	0.705	Nose running (rhinorrhea)	0.812	–
	Bones and muscle aches	0.682	Perspiring	0.534	–
	Gooseflesh	0.636	Hot flashes	0.501	–
	Shaking	0.633	–	–	–
	Cold flashes	0.622	–	–	–
	Restless	0.620	–	–	–
	Anxious	0.608	–	–	–
	Feel like using	0.602	–	–	–
	Muscle twitching	0.578	–	–	–
Clinical Opiate Withdrawal Scale (COWS)	Gastrointestinal upset	0.725	Pupil size	0.700	Sweating
	Bone or joint aches	0.716	Runny nose or tearing	0.638	Pulse
	Anxiety or irritability	0.712	–	–	
	Tremor	0.670	–	–	
	Gooseflesh skin	0.637	–	–	
	Restlessness	0.581	–	–	
	Yawning	0.528	–	–	

Factors based upon principal component analysis with Varimax rotation using data collected from time of peak severity report.

The COWS PCA (Table 2) conducted using symptoms at the time of peak report for each individual was also significant [*X*^2^(55) = 190.5, *p* < 0.001] and demonstrated reasonable sampling adequacy (0.702), with two factors identified and 9 of the 11 symptoms loading onto a factor. Factor 1 explained 28.5% of the variance and included the following seven symptoms: gastrointestinal upset, tremors, restlessness, anxiety or irritability, bone or joint aches, gooseflesh, and yawning. Factor 2 explained 12.4% of the variance and included the following two symptoms: pupil size and rhinorrhea/lacrimation. Two symptoms (resting pulse rate and sweating) did not satisfactorily load on any factor. A repeated measures ANOVA revealed a significant main effect of factor [*F*_(1, 79)_ = 78.2, *p* < 0.001, eta^2^ = 0.50] and time [*F*_(7, 553)_ = 27.5, *p* < 0.001, eta^2^ = 0.26] though the interaction was not significant (*p* = 0.17). Both factors demonstrated a significant increase from baseline (*p* < 0.05) at each of the timepoints (see Figure 3).

## 4. Discussion

These analyses sought to establish an operational definition of precipitated opioid withdrawal. Results revealed that an increase in 10 points on the SOWS (or 15% change in score) or an increase in 6 points on the COWS (or 12% change in score) that occurred within 1 h of administration was identified by participants as reflecting a clinically significant increase in the severity of their withdrawal experience as rated by both Bad Effects and Sick VAS. Moreover, analyses suggested that self-reported increase in symptoms of runny eyes, yawning, runny nose, sweating, hot flashes, and observed changes in pupil size and rhinorrhea/lacrimation may be uniquely associated with precipitated withdrawal; these could be explored as possible sentinel symptoms. It may also be useful to consider elevations on any one of these factors as an early warning sign of precipitated withdrawal, as opposed to requiring elevations on all loaded items, at which point withdrawal may already be progressing from mild to moderate severity.

The SOWS and COWS are two widely used measures of opioid withdrawal assessment. The present analyses build upon the severity thresholds (e.g., mild, moderate, and severe) established for these measures by defining precipitated withdrawal as a change from a non-zero level of withdrawal at baseline. As a result, the definition proposed here would complement these assessments that have traditionally been used to evaluate spontaneous opioid withdrawal severity. Moreover, identifying that a change in withdrawal corresponded to a 15% (SOWS) or 12% (COWS) change from baseline permits exploration of these definitions for other opioid withdrawal scales. Altogether this strategy should provide a method for identifying precipitated opioid withdrawal even in the context of spontaneous withdrawal, providing a pathway to begin differentiating withdrawal syndromes.

The distinction between spontaneous and precipitated withdrawal is important in clinical settings wherein patients are being inducted onto the MOUDs naltrexone and buprenorphine in the context of recent opioid agonist exposure. The ability to delineate these syndromes has also become more important given recent evidence that buprenorphine may be precipitating withdrawal in people who present with fentanyl exposure within the past 48 h (despite expressing moderate–severe spontaneous withdrawal) (11, 12). Before the proliferation of fentanyl, induction to naltrexone and buprenorphine was well-described, yet the interactions between fentanyl and naltrexone or buprenorphine are not well-understood, and revised induction methods are still being developed. Understanding when the precipitated withdrawal is occurring will help providers decide whether to cease or reduce MOUD doses (13) (perhaps to avoid additional precipitation) or to increase or accelerate MOUD doses (14, 15) (perhaps to surmount the precipitation) in the context of new-onset withdrawal; two strategies that are currently being investigated in the context of fentanyl exposure.

These data provide an initial operational definition and serve as an important foundation for understanding more about how the precipitated opioid withdrawal syndrome may vary from the spontaneous withdrawal syndrome. Laboratory studies that could examine known precipitated withdrawal using dose-dependent designs would provide a valuable test and opportunity to further refine these definitions. It would also be important to examine the validity of the precipitated withdrawal definitions in clinical settings following MOUD inductions, particularly among people with recent fentanyl exposure who may have clearer evidence of precipitated withdrawal. Additional efforts to determine whether a measure focused explicitly on the detection of precipitated withdrawal and that discerns precipitated from spontaneous withdrawal, perhaps composed of the aforementioned sentinel symptoms, may also be warranted.

These analyses have clear limitations. First, the study was not prospectively designed to develop a definition of precipitated withdrawal, and the granularity of assessments, as a result, is somewhat crude (every 15 min). It may be possible to determine a more precise onset of withdrawal expression with more frequent sampling. The degree to which these results will generalize outside of naloxone is also uncertain, and the study also does not provide an opportunity to differentiate between spontaneous and precipitated withdrawal syndromes outside of the challenge session. This will be an important next step for this line of research. In addition, MCID evaluations would ideally compare SOWS and COWS ratings against a gold standard and a validated measure of precipitated withdrawal severity vs. the global VAS ratings used here. Unfortunately, no such measure exists in the OUD field, so this study chose to anchor the MCID against two VAS scales that captured global experiences (vs. symptom-driven changes that were restricted to the symptoms being queried) and which were systematically determined to have statistically different categories. These categories should be examined further to verify the adequacy of the categorical distributions. Finally, the sample may have been underpowered for the analyses conducted.

## 5. Conclusion

These data provide an empirically informed strategy for operationally defining precipitated opioid withdrawal as an increase in 10 (15% MPE) or 6 (12% MPE) points on the SOWS and COWS, respectively, within 60 min of dose administration. Self-reported increase in eyes tearing, yawning, runny nose, perspiring, hot flashes, and observed increase in pupil size and rhinorrhea/lacrimation were also associated with an onset of precipitated withdrawal and could be useful symptoms to monitor. While these outcomes should be considered preliminary, they provide an important path for future exploration of precipitated opioid withdrawal. This research is crucial for both empirical progress in the context of fentanyl to buprenorphine inductions and future studies to help providers differentiate between spontaneous and precipitated withdrawal. Ultimately these data may be useful for advancing our understanding of the prevalence of this problem and identifying solutions to ease the induction process for providers and patients.

## Data availability statement

The raw data supporting these conclusions can be made available upon direct request to the authors.

## Ethics statement

The studies involving human participants were reviewed and approved by Johns Hopkins University Institutional Review Board. The patients/participants provided their written informed consent to participate in this study.

## Author contributions

ES received funding for the parent study, which was executed and completed with KD. KD wrote the first draft of the manuscript and led statistical analyses. All authors contributed to study manuscript and data interpretation.
